# Characterization and visualization of RNA secondary structure Boltzmann ensemble via information theory

**DOI:** 10.1186/s12859-018-2078-5

**Published:** 2018-03-05

**Authors:** Luan Lin, Wilson H. McKerrow, Bryce Richards, Chukiat Phonsom, Charles E. Lawrence

**Affiliations:** 1 Center for Devices and Radiological Health, U.S. Food and Drug Administration, Silver Spring, 20993 MD USA; 2 Division of Applied Mathematics, Brown University, Providence, 02912 RI USA; 3 Software Engineer, Google, New York, 10011 NY USA; 4 Department of Mathematics, University of Southern California, Los Angeles, 90089 CA USA

**Keywords:** RNA, RNA secondary structure, Nearest neighbor model, Information theory, Mutual information

## Abstract

**Background:**

The nearest neighbor model and associated dynamic programming algorithms allow for the efficient estimation of the RNA secondary structure Boltzmann ensemble. However because a given RNA secondary structure only contains a fraction of the possible helices that could form from a given sequence, the Boltzmann ensemble is multimodal. Several methods exist for clustering structures and finding those modes. However less focus is given to exploring the underlying reasons for this multimodality: the presence of conflicting basepairs. Information theory, or more specifically mutual information, provides a method to identify those basepairs that are key to the secondary structure.

**Results:**

To this end we find most informative basepairs and visualize the effect of these basepairs on the secondary structure. Knowing whether a most informative basepair is present tells us not only the status of the particular pair but also provides a large amount of information about which other pairs are present or not present. We find that a few basepairs account for a large amount of the structural uncertainty. The identification of these pairs indicates small changes to sequence or stability that will have a large effect on structure.

**Conclusion:**

We provide a novel algorithm that uses mutual information to identify the key basepairs that lead to a multimodal Boltzmann distribution. We then visualize the effect of these pairs on the overall Boltzmann ensemble.

## Background

RNA plays an important role in many biological processes, and next generation sequencing technologies have revealed a large number of novel non-coding RNA transcripts whose roles in biological processes are only beginning to be understood. Because the structure of macromolecules is often key to their function, the discovery of RNA structure has become increasingly important. While much progress has been made in the experimental determination of RNA structure, the disparity between RNA structure and sequence has continued to grow [1]. Thus computational tools that illuminate the physics of RNA structure are as important as ever.

Because secondary structure (SS) provides by far the largest contribution to the overall stability of an RNA molecule and precedes 3-D contact formation in the folding process, algorithms for the prediction of RNA SS continue to be an important component of structural prediction [2]. RNA SS algorithms have been developed for the prediction of structure from multiple related sequences [3, 4] and for SS prediction from a single RNA sequence. Here we focus on the latter class. The most popular RNA SS algorithms use recursive dynamic programming methods based on nearest neighbor energy calculations: to find the minimum free energy (MFE) structure [5–7]; to find the partition function [8]; to sample from the Boltzmann weighted ensemble [9] and to predict structures from the Boltzmann ensemble [10, 11].

However despite the progress that has been made, prediction of RNA SS from a single sequence remains challenging, especially for longer sequences. Many RNA structures are bistable, forming different structures in different contexts. Others form pseudoknots: structural features that are excluded from standard RNA secondary structure prediction methods. But even for sequences with a single, known native structure containing no pseudoknots, the Boltzmann distribution is rarely unimodal. This has led to efforts to find clusters of structures when no single representative structure exists. Methods include standard clustering algorithms [11–13] and strategies tailored to RNA SS: RNAshapes [14–16] finds structures that share a common “shape”, and Rogers et al. [17] group structures that share common helices in a process called “profiling”. Both of these strategies simplify the RNA folding problem by abstracting from individual basepairs to the helices that define RNA SS. While grouping structures based on common features does allow for a simplified description, such methods do not provide insight into the underlying features – conflicting basepairs – that drive multimodality in the Boltzmann distribution. Identifying these conflicting pairs will provide insight into how these alternate structures interact.

Recent work on so called “riboSNitches” has shown that many SNPs in noncoding sequences have wide ranging and potentially disease inducing effects on RNA structure [18–20]. The presence of disease associated variants in noncoding regions highlights the need to understand the relationship between sequence variation and RNA SS [20]. However predicting potential riboSNitches remains difficult [21]. Finding a few basepairs that are key to the secondary structure indicates that a mutation preventing the formation of one of these pairs will send the structure into an alternate conformation with potentially harmful effect. Furthermore, for some RNAs, such as viral genomes, alternative structures are necessary for proper function [22]. Even if the alternate conformations differ widely, the differences can often be reduced to the presence or absence of a few pairs. Finding these pairs provides an insight into how the transition between conformations is controlled. Finally, the rapid folding of an RNA into its native structure requires avoiding kinetic traps [23]. Thus, identifying key conflicting basepairs indicates which pairs must be avoided and which pairs must form in order for an RNA to fold quickly.

We employ information theory, or more specifically mutual information to find these key conflicting basepairs. Information entropy has been used to measure the complexity of the Boltzmann ensemble [24, 25], and the mutual information between aligned sequences has been used to construct a consensus sequence [26, 27]. However less focus has been given to the mutual information between the basepairs of a single RNA molecule. Using the nearest neighbor model (excluding pseudoknots), as implemented in the RNAstructure package [28], our algorithm finds the basepairs that provide the most information about other basepairs: the most informative base pairs (MIBPs). We then visualize the effect of these pairs by plotting the marginal basepairing probabilities conditioned on the presence or absence of the MIBPs.

## Methods

### Nearest neighbor model

An RNA secondary structure (SS) is a string of bases (A, C, G, or U), called the sequence, together with a set of basepairs between non-adjacent letters. Basepairs are two element sets, where {*i*,*j*} denotes a pair between the *i*^*th*^ and *j*^*th*^ bases. For 1≤*i*<*j*≤*n*, *X*_*ij*_ is a random variable that is 1 when the {*i*,*j*} pair is present and 0 when it is not. Only Watson-Crick (A-U, G-C) and wobble (G-U) pairs are considered. The space of allowable secondary structures is constrained by the following two requirements:

1. (No triples): $\sum _{j} X_{ij} \leq 1$ for all *i*.
2. (No pseudoknots): *X*_*ij*_+*X*_*kl*_≤1 for all *i*<*k*<*j*<*l*.

**Fig. 1:**
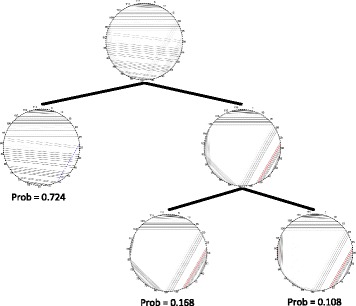
Visualization of *Tremella encephala* 5s rRNA (5s3_201 in the test set). Nucleotides are arranged around the edge of a circle and basepairs are drawn as chords connecting the paired bases. MIBPs that are constrained to be present are highlighted in red. Those that are absent are highlighted in blue. The darkness of a plotted basepair is proportional to its probability

If these requirements prevent two basepairs from existing simultaneously, we say that they conflict. Namely {*i*,*j*} and {*k*,*l*} conflict if *i* or *j* equals *k* or *l*, if *i*<*k*<*j*<*l*, or if *k*<*i*<*l*<*j*. If we draw basepairs as lines through a circle as in Fig. 1, conflicting pairs intersect on or inside the circle.

The free energy of a structure is given by experimentally derived parameters detailing the stability of various configurations of helices, loops and bulges. The Boltzmann probability is then proportional to the exponent of the negative free energy. In this paper we use the RNAstructure software [28] to calculate Boltzmann probabilities and to sample structures directly from this distribution.

### Most informative basepair

We equate the complexity (or simplicity) of a distribution with how unsure we are about the value of the corresponding random variable. The uncertainty in a basepair {*i*,*j*} is measured using information entropy:

1 $$ H[X_{ij}] = -p_{ij} \log_{2} p_{ij} - (1-p_{ij})\log_{2}(1-p_{ij})  $$

where *p*_*ij*_=*P*(*X*_*ij*_=1), 0 log0=0 and the units of *H* are in bits. When we are less sure about a basepair, *p*_*ij*_ is closer to 1/2 and *H*[ *X*_*ij*_] is larger. Conversely when we are more sure about a basepair, *p*_*ij*_ is closer to 0 or 1 and *H*[ *X*_*ij*_] is smaller.

Now if we condition on another basepair *X*_*kl*_, we have two conditional distributions: *P*(*X*_*ij*_|*X*_*kl*_=1) and *P*(*X*_*ij*_|*X*_*kl*_=0), each with corresponding entropies: *H*[ *X*_*ij*_|*X*_*kl*_=1] and *H*[ *X*_*ij*_|*X*_*kl*_=0]. The conditional entropy is defined to be

2 $${} H[\!X_{ij}|X_{kl}] = (1-p_{kl})H[\!X_{ij}|X_{kl}=0] + p_{kl} H[\!X_{ij}|X_{kl}=1]  $$

and is the average uncertainty in *X*_*ij*_ after we learn the value of *X*_*kl*_. Therefore the amount of information that *X*_*kl*_ tells us about *X*_*ij*_ is

3 $$ I(X_{ij};X_{kl}) = H[\!X_{ij}] - H[\!X_{ij}|X_{kl}]  $$

This value is referred to as the mutual information between *X*_*ij*_ and *X*_*kl*_ and it is symmetric [29]. By Eqs. 2 and 3, on average the distribution of *X*_*ij*_ conditioned on *X*_*kl*_ is *I*(*X*_*ij*_;*X*_*kl*_) bits simpler than the unconditioned distribution. We can then measure the amount of information that a basepair provides about the rest of the secondary structure by adding up its mutual information with each other basepair. We can then condition on the basepair that has the greatest sum of mutual information to get a less complex conditional distribution. We call this basepair the most informative basepair:

#### **Definition 1**

The most informative basepair (MIBP) is the basepair that has the largest sum of mutual information:

$$\text{MIBP} = \underset{kl}{\arg\!\max} \sum_{ij}I(X_{ij};X_{kl}) $$

Calculating mutual information requires the joint probability of every pair of basepairs, a computationally intensive task. However we can quickly estimate the mutual information from sampled structures. Structures can be sampled from the Boltzmann ensemble for a sequence of length *n* in $\mathcal {O}\left (n^{3}\right)$ time using RNAstructure or a similar tool. We can find the MIBP from sampled structures as follows:



Basepairs that appear in fewer than 10 or more than 990 samples will have low entropy and so will not make a significant contribution to mutual information. Thus we can improve computational efficiency without sacrificing accuracy by ignoring them. In general we find that 1000 samples is enough to get an accurate estimate of base pairing probabilities.

### Tree based clustering

We greedily employ the MIBP algorithm to build a binary tree that clusters structures based on the presence of MIBPs. We first split the space into a cluster that includes the MIBP and one that does not. We then find the conditional MIBP in each of the clusters and split those clusters in two. We continue this process until the product of the cluster probability and estimated mutual information falls below 2 bits.



The algorithm creates a binary tree of nested clusters, where each branch corresponds to conditioning on the presence or absence of a particular MIBP. The leaves of this tree are then an exhaustive set of clusters. An html file is created that draws the tree and plots the marginal basepairing probabilities at each node. See the “Results” section for examples. Algorithms that use mutual information to create binary trees, such as ID3 and C4.5 [30], are used widely in classification problems. This algorithm employs a similar concept, but it uses the mutual information between basepairs as there is no natural labelling for RNA secondary structures as would be the case in a classification problem.

### Conflicting basepairs and other cluster calculations

Once we have found MIBPs and divided the space, we can examine the individual clusters. First we look for basepairs that conflict with the MIBP. For each MIBP split, we first find the basepair that conflicts with the MIBP and is most probable. Because the MIBP and the conflicting pair cannot be present in the same structure, the most probable conflicting pair is also the conflicting basepair that has the most pairwise mutual information with the MIBP (see “Additional methods” section). We then continue in a greedy fashion, repeatedly finding the most probable basepair that conflicts with the MIBP and all previous conflicting pairs. We stop once we have found a total of five conflicting pairs. When conflicting pairs are present, they provide an explanation for the presence of divergent clusters.

We can also use RNAstructure to calculate the marginal probability of each basepair in each cluster and use that information to calculate the conditional entropy in each cluster. Finally, we can calculate the number of structures in each cluster by setting the free energy, *E*(*x*), equal to 0 for all structures *x* and then calculating the partition function. Note that RNAstructure counts structures with the same basepairs but different coaxial stacking separately.

## Results

To test our algorithm, we used a set of sequences and corresponding native structures provided to us by David Mathews. This data is used by the Mathews group to test the RNAstructure software package. The sequences are compiled from the following publications: [31–40]. The test set includes sequences from ten families whose secondary structures have been verified by comparative analysis: 5s, 16s and 23s rRNA, group 1 and 2 introns, RNAse P (RNAp), signal recognition particle (SRP), telomerase, tmRNA and tRNA. The 16s and 23s rRNA sequences were divided into four and six folding domains respectively [32, 33] to make the computation more tractable. For 5s, 16s, SRP, telomerase, tmRNA and tRNA, we considered only 10 randomly selected sequences from each family as the test set included a large number of sequences from these families. A list of all sequences considered can be found at the visualization site described in the next section.

### Visualizations

Visualizations of the Boltzmann ensembles of these sequences can be found at http://ccmbweb.ccv.brown.edu/wmckerro/MIBP/ Visualizations should be viewed in the firefox browser. Longer sequences may be slow to load. The visualizations draw the binary tree described in the “Methods” section. Clicking on a node in the tree reveals the conditional probability of each basepair in the corresponding cluster, showing how the presence or absence of MIBPs affects the structure. Figure 1 shows the circle diagrams arranged in a tree for an example RNA molecule.

### Entropy reduction

**Fig. 2:**
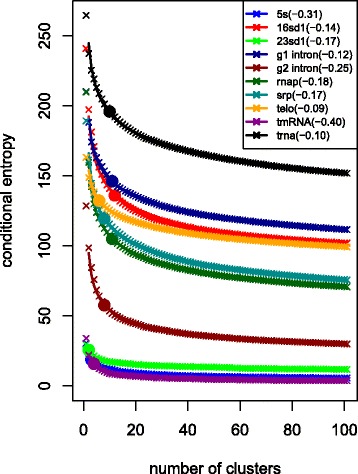
Entropy as a function of number of clusters for one sequence from each of the ten families. Power law functions of the form *y*=*ax*^*b*^ are estimated by linear least squares regression after log-log transform and plotted as lines. The value of *b* is given parenthetically. The two bit cutoff is highlighted by a filled circle

To see how adding new clusters affects the conditional entropy, we ran our algorithm for 100 steps on one sequence from each family, yielding 101 clusters for each sequence. As a function of the number of clusters, the entropy closely follows a power law with an exponent that varies from -0.1 for the *Chinchilla brevicaudata* telomerase (AF221937.99-545 in the test set) and trna sequences to -0.4 for the *Clostridium perfringens* tmRNA sequence (Clos.perf._CP000246_1-361). (See Fig. 2.)

**Fig. 3:**
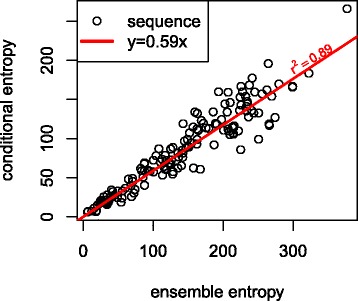
Ensemble entropy vs conditional entropy. Running the MIBP algorithm with a 2 bit cutoff yields an entropy reductions of nearly a half. For example the Chlamydomonas 5s rRNA (5s_13 in the test set) has an ensemble entropy of 49 bits, but after conditioning on the MIBPs, only 25.5 bits of uncertainty remains. Each point represents one of the sequences from one of the ten families described at the beginning of the results section

We also test how the default 2 bit cutoff (see “Methods” section) affects the conditional entropy. Across all the seqeunces tested, the 2 bit cutoff yielded an average of 3.4 clusters with average entropy reduction of nearly a half (See Fig. 3). This reduction is slightly larger than would be predicted by the power law because the first couple splits often provide greater entropy reduction. It would be possible to use a smaller cutoff, yielding more clusters, but the power law functions indicate that this would likely yield only a modest decrease in ensemble entropy.

### Entropy constraints and number of structures

**Fig. 4:**
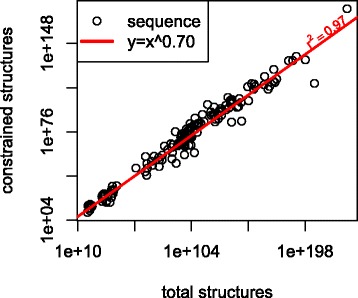
Number of structures before and after basepairs with entropy less than 0.002 bits are constrained. Constraining basepairs reduces the orders of magnitude by about one fourth. Each point represents one of the sequences from one of the ten families described at the beginning of the results section

Constraining basepairs with low entropy excludes most of the possible structures, but retains most of the probability mass. This is consistent with concentration of measure phenomena often seen in high dimensional probability distributions [41]. Basepairs with entropy less than 0.002 bits were constrained to be unpaired if they have probability near 0 and paired if they have probability near 1. For every sequence, the entropy constraints removed less than 5% of the probability mass but resulted in about a one fourth reduction in the orders of magnitude for the total number of possible structures. For a short sequence such as *Spirocodon saltatrix* 5s rRNA (5s3_220) this means a reduction of 8 orders of magnitude from 4×10^31^ to 6×10^23^. However for a longer sequence such as a *Saccharomyces cerevisiae* group II intron (ya1) the entropy constraints result in a reduction of almost 50 orders of magnitude: from 1×10^172^ to 9×10^126^ (see Fig. 4).

### Cluster with native structure

Consistent with previous observations [11] the fact that a cluster contains more probability does not necessarily mean that it will contain the native structure. In fact, for the sequences tested, the probability of the native cluster is not significantly larger than the probability of a cluster chosen uniformly at random (permutation *p*-value = 0.47). This is likely due to the fact that secondary structure prediction algorithms struggle to provide accurate predictions for some of the longer sequences considered. If we rerun the analysis on the 309 5s RNA sequences in the RNAstructure test set, we find that the average native cluster size is 41.5% – significantly higher than the mean random cluster size of 37.0% (permutation *p*-value = 0.02). However it is still far short of the mean expected cluster size of 51.9%. This indicates that, at least for smaller sequences, the native structure is more likely to be found in a higher probability cluster, but that it less likely to be found in such a cluster than the Boltzmann ensemble indicates. Permutation tests were done in R using the coin package with 10,000 samples.

### Conflicting basepairs

We find that many MIBPs are part of a pair of conflicting basepairs, but we also find that in many cases the MIBP is part of a set of more than two mutually conflicting basepairs. Each pair in the set of mutually conflicting pairs is somewhat probable on its own, but due to the no pseudoknots and no triples constraints, only one can be present in a given structure.

For 40% of MIBPs, the MIBP represents a binary choice between two basepairs. In such cases there is a conflicting base that is present in at least 90% of sampled structures that do not include the MIBP. In other cases the MIBP is one choice among a set of mutually conflicting pairs. For 84% of MIBPs, 90% of samples that do not include the MIBP include one member of a set of up to five basepairs that conflict with each other and the MIBP.

### Mutations to the MIBP nucleotides

**Fig. 5:**
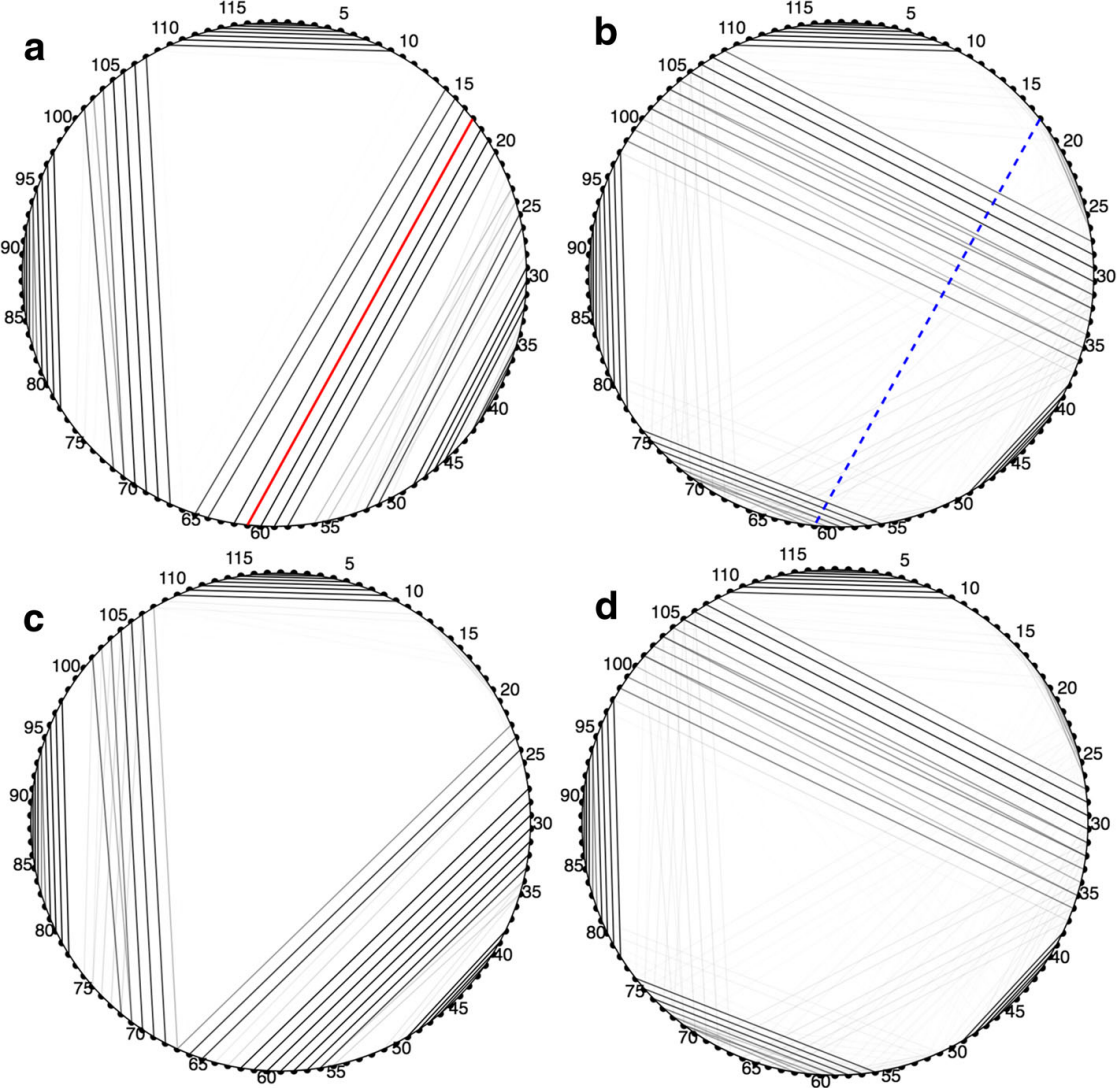
Mutating the ends of the MIBP or conflicting pair has a large effect on the resulting RNA SS. **a** Basepair probabilities conditioned on the presence of MIBP. **b** Basepair probabilities conditioned on the absence of MIBP. **c** Basepair probabilities when conflicting pair is mutated. **d** Basepair probabilities when MIBP is mutated. 5s rRNA from the freshwater alga *Hydrurus foetidus* (5s3_71 in the test set) is the sequence considered

In this subsection we consider a 118 nucleotide 5s rRNA from the freshwater alga *Hydrurus foetidus* (5s3_71 in the test set). The MIBP algorithm finds one most informative basepair – (17,61) – dividing the Boltzmann space into two classes. 82% of structures that do not contain the MIBP contain the conflicting pair (29,107). This implies that mutating the sequence so that one of these two basepairs cannot form would bias the structure to fall into one class over the other. Indeed, editing the 17th nucleotide from a C to a A yields a Boltzmann distribution that is similar to conditioning on the absence of the MIBP. Editing the 29th position from a C to a G yields a structure that is similar to conditioning on the presence of the MIBP. However a different set of basepairs constitute one of the helices (see Fig. 5).

### Mutual information and RiboSNitches

Woods and Laederach [42] use SHAPE data to classify mutations into three categories based on whether they cause (i) “no differences or small differences”, (ii) “local differences”, or (iii) “global differences” to the RNA secondary structure. We focus on one of the sequences considered by Woods and Laedarach: a 16s rRNA 4 way junction (16SFWJ_1M7_0001 in RMDB: https://rmdb.stanford.edu/). While the ends of the MIBP (146,216) are not mutated, nearby positions that are likely to form a helix with the MIBP are mutated: a G to C mutation at position 125 causes local differences, and C to G mutations at positions 214 and 221 causes global differences. Most of the mutations considered (74%) cause little or no difference to the RNA SS. The third mutation that affects global change, a G to C mutation at position 177, forms the conflicting pair for one of the three additional MIBPs found with the standard 2 bit cutoff.

**Fig. 6:**
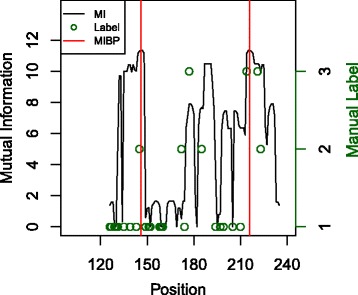
Mutual information sum and label assigned by Woods and Laederach [42] for the 16s rRNA four way junction (16SFWJ_1M7_0001 in RMDB: https://rmdb.stanford.edu/). A label of 1 indicates that the mutation causes little or no change in RNA SS. A label of 2 indicates that the mutation causes significant but primarily local changes to the RNA SS. A label of 3 indicates that the mutation causes global changes to the RNA SS

We also compare the mutation category to the maximum sum of mutual information for a basepair originating from the mutated position. The mean mutual information for mutations that cause global changes in RNA SS (9.85 bits) is greater than the mean MI for positions local changes (7.36 bits) and much greater than the mean MI for positions that cause little or no change (3.06 bits). Figure 6 shows the mutual information at all the mutated positions. The html visualization for this molecule can be accessed at: http://ccmbweb.ccv.brown.edu/wmckerro/MIBP/16SFWJ_1M7_0001.html.

### A viral RNA with a key alternate conformation

**Fig. 7:**
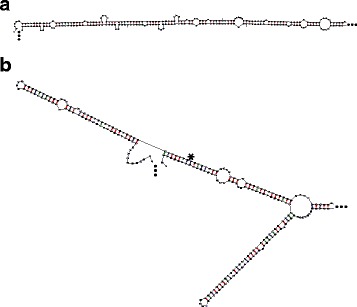
Structure predictions for Hepatitis Delta Virus Genotype III. **a** Without MIBP, a rod shaped structure forms. **b** With the MIBP, the branched structure described in [43] forms. The edited position is indicated with an asterisk. Structures were drawn using the mfold webserver [49]

Hepatitis Delta Virus (HDV) normally adopts a rod shaped configuration, but HDV Genotype III must also form a branched structure in order to undergo an essential RNA editing event [43]. We ran our MIBP algorithm on a section of the HDV Genotype III (reverse complement of GenBank: HF679406.1, nucleotides 499-1097). Most sampled structures form a rod shaped structure (Fig. 7a), but some form the branched structure described in [43] (Fig. 7b). The MIBP algorithm shows that the branched structure forms when the MIBP – (1020,1086) – is present and the rod structure forms when it is absent.

### SHAPE

The MIBP algorithm can also be used to show how the inclusion of experimental data, such as SHAPE (selective 2’-hydroxyl acylation analyzed by primer extension) [44] affects the probability distribution. SHAPE data is used by RNAstructure to calculate a “pseudoenergy”: *E*^∗^(*X*)=*E*(*X*)+*C*(*X*,*D*) where *C*(*X*,*D*) is a “pseudoenergy change term” that reflects how well the structure *X* fits the experimental data *D* [45]. Since Boltzmann probability is calculated by exponentiating the free energy, this is equivalent to using the nearest neighbor model as a Bayesian prior and then updating it with a likelihood term calculated from the SHAPE data. The data we use is from [46] and [45].

**Fig. 8:**
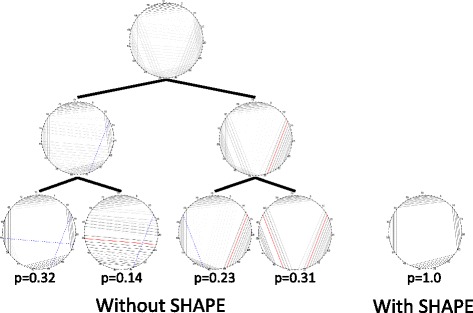
Visualization of *E. coli* tRNA (Phe) Boltzmann space with and without SHAPE data. Nucleotides are arranged around the edge of a circle and basepairs are drawn as chords connecting the paired bases. MIBPs that are constrained to be present are highlighted in red. Those that are absent are highlighted in blue. The darkness of a plotted basepair is proportional to its probability. The native structure forms a clover-leaf shape as in the left most cluster and the cluster with SHAPE data

The inclusion of SHAPE data yields a simpler distribution with fewer conflicting pairs and samples that are more similar to the native structure. When SHAPE data is included, entropy is lower by a factor ranging from 2 to 17.3. On average the expected difference between a sampled structure and the native structure measured in basepairs different decreases by a factor of 9.6. Figure 8 shows a visualization of the distribution with and without SHAPE data for the most dramatic example – a phenylalanine tRNA. In two cases the inclusion of SHAPE yields a distribution in which no basepair has at least 2 bits of mutual information. For the other four sequences, the largest cluster contains the native structure. This is only true for two of the six sequences without SHAPE data “See Table 1”.

**Table 1:** Summary statistics for six sequences, with and without shape

Sequence	Length	Clusters	Entropy	Conditional entropy	Mean error
Without SHAPE data					
Riboswitch	71	3	25.3533	13.3428	5.5875
5s rRNA	120	4	51.293	30.8405	53.3667
P546	155	7	114.5027	54.0569	44.1121
RNase P	154	8	86.7581	39.7549	36.8573
HCV	95	5	37.893	11.3547	19.7718
tRNA (Phe)	76	4	52.1349	17.404	20.474
With SHAPE data					
Riboswitch	71	1	4.9419	4.9419	1.1114
5s rRNA	120	3	21.376	11.5638	10.1141
P546	155	3	28.1101	16.4472	7.1615
RNase P	154	3	20.3484	11.4831	20.9432
HCV	95	2	18.6929	1.7029	6.4995
tRNA (Phe)	76	1	3.0043	3.0043	0.5634

Mean error is the expected number of basepairs that differ between the native structure and a sampled structure

## Discussion

Our algorithm provides a characterization of the Boltzmann weighted space by iteratively dividing the secondary structure space based on the presence or absence of MIBPs. Mutual information allows us to find a small number of basepairs that account for about half of the uncertainty in the Boltzmann ensemble. We can then visualize the ensemble distribution as a finite mixture of a small set of simpler distributions.

Our method differs from similar methods [14, 15, 17, 47] by focusing on the basepairs that cause the Boltzmann distribution to be multimodal. Our method not only groups similar structures, but also identifies the most informative basepairs that determine which group a structure falls into. The RNA profiling method [17] does provide a branching set of helices for each class. However using mutual information we are able to condense that set of helices into a few key most informative basepairs. While two alternate structures may include very different sets of helices, it is often the case that one need only affect the stability of a single most informative basepair to bias one alternate structure over another.

With the realization that point mutations can change the secondary structure of an RNA transcript enough to cause misregulation and disease, there is a need to understand how SNPs affect RNA SS [18, 20]. MIBPs show that while alternate structures may have little overlap in shape or basepairing, it is often the case that constraining a single basepair is enough to bias one structure over another. Thus if a mutation disrupts the MIBP or its conflicting pair, it is likely to cause a global change in RNA SS. Indeed we find that mutations at positions with high mutual information are likely to have wide ranging effects on structure. Furthermore as new methods to edit specific sites in an RNA molecule emerge [48], the need for tools that can connect nucleotide changes to structure will only increase.

Finding MIBPs, provides valuable insight into the formation of alternate structures. The viral genome of HDV Genotype III must adopt an alternate conformation in order to undergo a key RNA editing event [43]. Our algorithm shows that the conformation can be predicted by the presence or absence of a single MIBP. This key basepair provide a starting place for understanding how and when this transition occurs.

Our results show that it only takes a few basepairs to encode half of the information present in a sample from the Boltzmann distribution. We also show that most possible basepairs have entropy near zero and are irrelevant to the Boltzmann distribution. If these low entropy pairs are constrained, the size of the structure space shrinks dramatically. Finally, characterizing the Boltzmann ensemble allows us to see how the incorporation of experimental data affects structure prediction.

The MIBP algorithm is not limited to the nearest-neighbor thermodynamic model. Most informative basepairs exist for any probabilistic model of RNA and can be calculated either from samples or from the joint distributions of pairs of basepairs. In particular this strategy can be applied with ease to any single sequence stochastic context free grammar model. The MIBP algorithm uses single basepairs as its features, when it is generally whole helices and other large structural features that define an RNA secondary structure. In most cases a single MIBP can be used a proxy for a whole helix, but this can make finding conflicting basepairs difficult when two conflicting helices partially overlap. Nevertheless, mutual information is not limited to basepairs. Our algorithm could be combined with a method that abstracts from basepairs to find a most informative feature of some kind.

## Conclusion

Most informative basepairs provide a novel method for exploring and visualizing the RNA secondary structure Boltzmann ensemble. Unlike other methods for characterizing the Boltzmann ensemble, the MIBP method provides a set of key basepairs that determine which structure will form from a given sequence. These pairs suggest that small changes either to the sequence or to the stability of specific pairs will bias a molecule to fold into one alternate structure over another.

## Additional methods

### **Claim 1**

For fixed *p*_*ij*_, if *X*_*ij*_ and *X*_*kl*_ conflict, then *I*(*X*_*ij*_;*X*_*kl*_) is a monotonic increasing function of *p*_*kl*_.

**Proof:** Using the formula for *I*(*X*;*Y*) given in [29] and the fact that *P*(*X*_*ij*_=1,*X*_*kl*_=1)=0:

$${{\begin{aligned} I(X_{ij};X_{kl}) &= (1-p_{ij}-p_{kl})\log_{2}\frac{1-p_{ij}-p_{kl}}{(1-p_{ij})(1-p_{kl})} \\ &\quad+ p_{ij} \log_{2} \frac{p_{ij}}{p_{ij}(1-p_{kl})} + p_{kl} \log_{2} \frac{p_{kl}}{p_{kl}(1-p_{ij})} \\ &= (1-p_{ij}-p_{kl})\log_{2}(1-p_{ij}-p_{kl})\\ &\quad- (1-p_{ij})\log_{2}(1-p_{ij}) - (1-p_{kl})\log_{2}(1-p_{kl}) \end{aligned}}} $$

Taking the derivative with respect of *p*_*kl*_ yields:

$$\begin{array}{*{20}l}{} \frac{d I(X_{ij};X_{kl})}{d p_{kl}} &= \frac{1}{\log 2}\! \left[ -2 \log (1-p_{ij}-p_{kl} + 2 \log (1-p_{kl}) \right ] \\ &= \frac{2}{\log 2} \left[\log \frac{1-p_{kl}}{1-p_{ij}-p_{kl}} \right ] > 0 \end{array} $$

Thus the claim is proved.

## Abbreviations

HDV: Hepatitis Delta Virus
MIBP: most informative basepair
MFE: minimum free energy
RNAp: RNAse P
rRNA: ribosomal rna
SHAPE: selective 2’-hydroxyl acylation analyzed by primer extension
SRP: signal recognition particle
SS: secondary structure
tmRNA: transfer-messenger RNA
tRNA: transfer RNA
